# Generation of an immortalised erythroid cell line from haematopoietic stem cells of a haemoglobin E/β-thalassemia patient

**DOI:** 10.1038/s41598-020-73991-4

**Published:** 2020-10-08

**Authors:** Kongtana Trakarnsanga, Chartsiam Tipgomut, Chanatip Metheetrairut, Methichit Wattanapanitch, Archrob Khuhapinant, Saiphon Poldee, Ryo Kurita, Yukio Nakamura, Chatchawan Srisawat, Jan Frayne

**Affiliations:** 1 Department of Biochemistry, Faculty of Medicine Siriraj Hospital, Mahidol University, Bangkok, Thailand; 2 Siriraj Center for Regenerative Medicine, Research Department, Faculty of Medicine Siriraj Hospital, Mahidol University, Bangkok, Thailand; 3 Division of Hematology, Department of Medicine, Faculty of Medicine Siriraj Hospital, Mahidol University, Bangkok, Thailand; 4 Department of Research and Development, Central Blood Institute, Blood Service Headquarters, Japanese Red Cross Society, Tokyo, Japan; 5 Cell Engineering Division, RIKEN BioResource Research Center, Ibaraki, Japan; 6 School of Biochemistry, University of Bristol, Bristol, UK; 7 NIHR Blood and Transplant Research Unit in Red Blood Cell Products, University of Bristol, Bristol, UK

**Keywords:** Haematopoietic stem cells, Stem-cell biotechnology

## Abstract

The β-thalassemia syndromes are the most prevalent genetic disorder globally, characterised by reduced or absent β-globin chain synthesis. HbE/β-thalassemia is a subtype of β-thalassemia with extremely high frequency in Asia. Studying molecular defects behind β-thalassemia is severely impeded by paucity of material from patients and lack of suitable cell lines. Approaches to derive erythroid cells from induced pluripotent stem cells (iPSCs) created from patients are confounded by poor levels of erythroid cell expansion, aberrant or incomplete erythroid differentiation and foetal/embryonic rather than adult globin expression. In this study we generate an immortalised erythroid cell line from peripheral blood stem cells of a HbE/β-thalassemia patient. Morphological analysis shows the cells are proerythroblasts with some early basophilic erythroblasts, with no change in morphology over time in culture. The line differentiates along the erythroid pathway to orthochromatic erythroblasts and reticulocytes. Importantly, unlike iPSCs, the line maintains the haemoglobin profile of the patient’s red blood cells. This is the first human cellular model for β-thalassemia providing a sustainable source of disease cells for studying underlying disease mechanisms and for use as drug screening platform, particularly for reagents designed to increase foetal haemoglobin expression as we have additionally demonstrated with hydroxyurea.

## Introduction

β-thalassemia is a common genetic disorder with high prevalence in areas where malaria is (or was) highly endemic. It can be caused by any of over 300 mutations in and around the β-globin gene, resulting in reduced or absent β-globin synthesis, a component of haemoglobin (Hb) in red blood cells (RBCs) required for oxygen delivery in the body. A particular subtype, HbE/β-thalassemia, with extremely high frequency in many countries in Asia^1^, is caused by compound heterozygous mutation of the β-globin genes. Mutation of the one allele causes reduction or absence of β-globin production (dependent on the mutation present), whilst the HbE mutation causes aberrant splicing of a proportion of transcripts along with a structural variant of the β-globin translated from the remaining normally spliced transcripts, with overall reduced levels of protein produced. In all cases of β-thalassemia reduced or absent expression of β-globin results in globin chain imbalance with excess α-globin forming insoluble aggregates which undergo auto-oxidation leading to increased ROS and a range of intracellular downstream events resulting in the observed disease pathophysiology^2^.

At present, treatment for most thalassemia patients is regular blood transfusions, with resultant consequences such as iron overload and transfusion reactions. Bone marrow transplant, a curative treatment, is limited to patients who have compatible stem cell donors, and is not an accessible therapy for many patients in low and middle-income countries where the disease is prevalent. Clinical advances have been achieved using autologous transplantation and β-globin gene transfer (reviewed by Dreuzy et al.^3^). However, such gene therapy is still largely experimental and expensive, with efficacy and safety issues being of major concerns (reviewed by Cappellini et al.^2^).

Individuals with concomitant hereditary persistence of foetal haemoglobin and β-thalassemia exhibit reduced disease severity^4^, due to reduction in the concentration of free α-globin subunits. Hence increasing the level of foetal haemoglobin (HbF) in patients has the potential to ameliorate clinical symptoms, and is a major focus of much ongoing research (reviewed by Cappellini et al.^2^). However, currently, much of the knowledge of erythropoiesis and red blood cell disorders, such as β-thalassemia is based on mouse models^5–7^. Although these studies are valuable, fundamental differences between mouse and human erythropoiesis are becoming more apparent^8–10^.

Erythroid cells can be differentiated in vitro from peripheral blood stem cells. However, the cells have a limited capacity for self-renewal with the total number of erythroid cells produced restricted by their expansion potential^11^. In order to generate sufficient quantities of cells for study, repeated collections of stem cells are therefore required, an inappropriate approach if requiring cells from anaemic patients. Thus, new approaches for studying β-thalassemia are essential for understanding the underlying molecular mechanisms, developing new therapeutics and creating screening platforms for the development of new drugs.

A number of reports have derived iPSCs from thalassemic patients^12,13^, however erythroid cells differentiated from iPSCs are confounded by poor levels of expansion, aberrant or incomplete erythroid differentiation and express predominantly foetal/embryonic rather than adult globin^14, 15^ making them unsuitable as model lines. We previously developed methodology and generated an adult human immortalised erythroid cell line (BEL-A) that recapitulates normal erythropoiesis, expresses normal levels of adult globin, terminally differentiates and enucleates to generate mature reticulocytes^16^. We have now taken this approach forward, creating the first immortalised line from a patient with HbE/β-thalassemia (Siriraj Bristol Beta-thalassemia/haemoglobin E cell line; SiBBE) providing a sustainable supply of cells as a disease model system.

## Results and discussion

### Generation of an immortalised HbE/β-thalassemia erythroid line (SiBBE)

**Figure 1:**
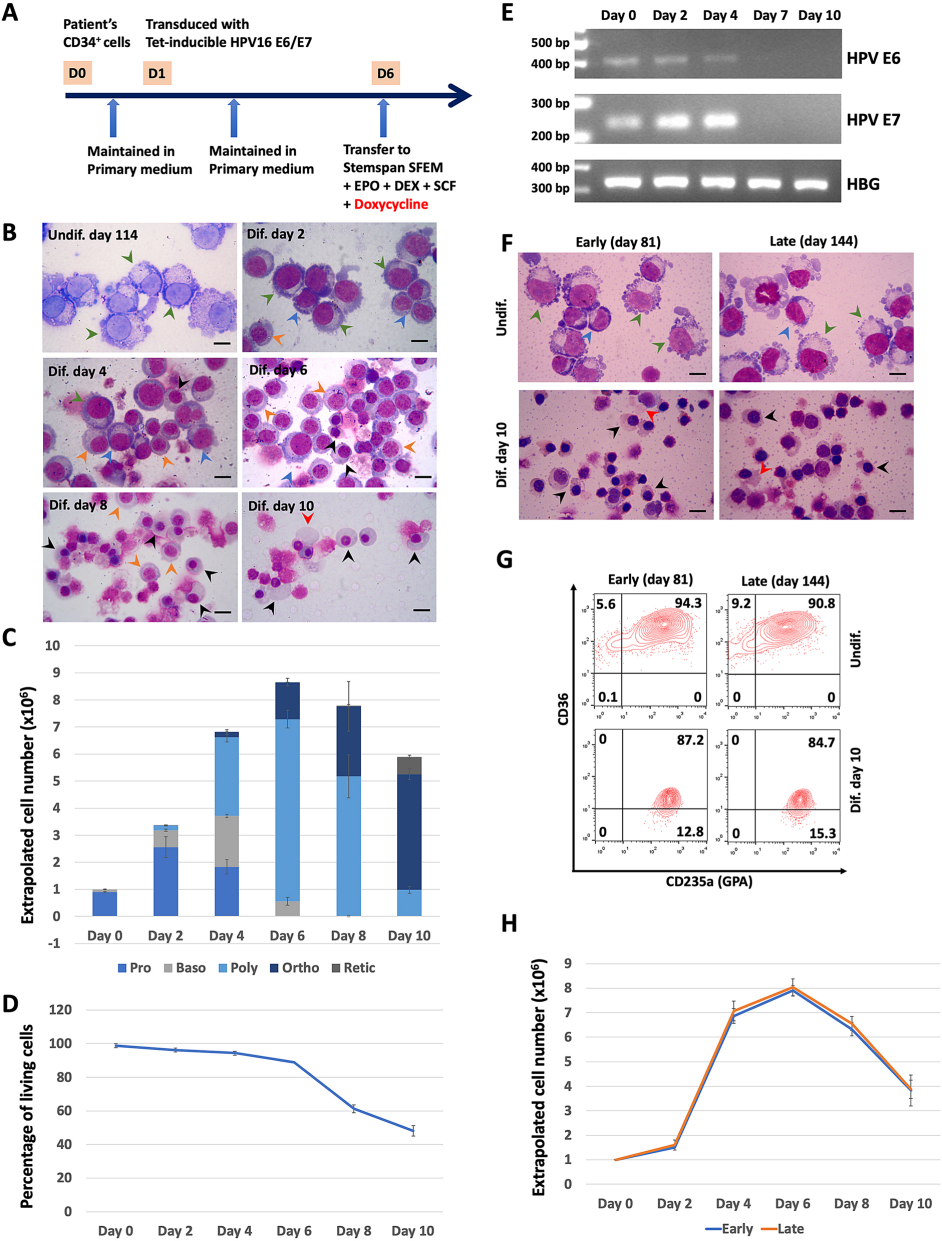
(**A**) A schematic diagram illustrating steps in the generation of the immortalised erythroid cell line (SiBBE) from stem cells collected from blood of a patient with haemoglobin E/β-thalassemia. (**B**) Undifferentiated erythroid cells (undif.) on day 114 in expansion medium and differentiated erythroid cells (dif.) on day 2, 4, 6, 8 and 10 in erythroid differentiation medium stained with Leishman reagent and analysed by light microscopy (scale bar = 10 μm). Green arrows = proerythroblasts; Blue arrows = basophilic erythroblasts; Orange arrows = polychromatic erythroblasts; black arrows = orthochromatic erythroblasts; red arrows = reticulocytes. (**C**) Extrapolated number of erythroid cell types at different time points during erythroid differentiation (mean ± SD, n = 3). (**D**) Percentage of living cells during erythroid differentiation determined by Trypan blue assay (mean ± SD, n = 3). (**E**) Expression of HPV16 E6, E7 and gamma globin (HBG) (control) determined by RT-PCR. Full-length gels are presented in Supplementary Fig. 3. (**F**) Undifferentiated early (day 81) and late (day 144) passage cells in expansion medium (undif.) and at day 10 in differentiation medium (dif. day 10) stained with Leishman reagent and analysed by light microscopy (scale bar = 10 μm). (**G**) Flow cytometric analysis of cells in (**E**) incubated with antibodies to glycophorin A (GPA; CD235a) and CD36. (**H**) Extrapolated cell numbers of early (day 77 to 81; blue) and late (day 136 to 144; orange) passage cells during erythroid differentiation (mean ± SD, n = 3).

Isolated patient CD34^+^ cells were transferred to primary medium of our erythroid culture system^17^ for 24 h, then transduced with a Tet-inducible HPV-E6/E7 construct^18^. On day 6 the cells were transferred to and maintained in expansion medium as described in Trakarnsanga et al.^16^, with cell counts and total medium changes every 2–3 days (schematic of protocol shown in Fig. 1A). The cell line was maintained in continuous culture for over 100 days, with a mean doubling time of 17 h. This is comparable to 18.7 ± 2.0 h for the normal erythroid cell line BEL-A^19^. After 100 days the line was well established with aliquots frozen for storage. Their ability to re-establish on thawing was confirmed. Morphological analysis showed the cells to be proerythroblasts with some early basophilic erythroblasts (Fig. 1B, undif., line age day 114). For further erythroid differentiation and maturation, the cells were transferred to primary medium of our erythroid culture system^17^ for 6 days, with doxycycline for day 1–4, followed by tertiary medium of the same system thereafter. Cells differentiated along the erythroid pathway with an enucleation rate of 10.8 ± 0.6% on day 10 (Fig. 1B,C; n = 3). Maximum fold expansion of 8.7 ± 0.4 fold was obtained on day 6 of differentiation (Fig. 1C; value calculated from number of cells transferred to differentiation medium). The number and viability of differentiated SiBBE cells decreased after day 6 of differentiation (Fig. 1C,D), the timing of cell death corresponding with the majority of the cells being polychromatic erythroblasts (Fig. 1C). This is in line with that observed for erythroid cells differentiated from β-thalassemia patient CD34^+^ cells which apoptose at the polychromatic stage due to ineffective erythropoiesis^20,21^. In contrast the normal erythroid cell line BEL-A cells decrease in number after day 8 due to death of orthochromatic erythroblasts that fail to enucleate^19^. The expression of HPV16 E6 and E7 was lost from the cells following removal of doxycycline on day 4 of differentiation (Fig. 1E).

There was no change in morphology of the cells over time in culture (Fig. 1F, day 81 and 144, undif.). This was confirmed by flow cytometry analysis of CD36 and glycophorin A^22^ levels for cells at line age day 77 to 81 and day 136 to 144 (Fig. 1G top panels, representative populations of early and late SiBBE passage shown). 95.4 ± 0.9% undifferentiated early cells were GPA^med/high^CD36^high^ compared to 93 ± 2.6% for the late population (p > 0.05; n = 3).

To confirm unchanged differentiation of SiBBE after prolonged expansion, early and late passage cells (as above) were transferred to differentiation medium. There was no significant difference in expansion kinetics of the early and late SiBBE cells during differentiation (Fig. 1H). At the end of differentiation (day 10), the early passage population was 17.4 ± 1.6% polychromatic erythroblasts, 72.6 ± 1.7% orthochromatic erythroblasts and 10.0 ± 1.1% reticulocytes, and the late passage population was 17.6 ± 1.4% polychromatic erythroblasts, 72.0 ± 1.6% orthochromatic erythroblasts and 10.4 ± 0.6% reticulocytes (p > 0.05; n = 3) (Fig. 1F, dif. day 10; representative images of cell morphology shown). Flow cytometry analysis of the differentiated cells on day 10 showed no significant difference between the levels of GPA and CD36 between the early and late passage populations, 86.7 ± 2.9% and 81.2 ± 3.1% of differentiated early and late populations were GPA^high^CD36^low^, respectively (p > 0.05; n = 3) (Fig. 1G, lower panels; representative flow plots shown). There was also no significant difference in enucleation rates, 10.0 ± 1.1% in the differentiated early cells compared to 10.4 ± 0.6% in the differentiated late cells (p > 0.05, n = 3). These data confirm no difference in behaviour or phenotype of SiBBE following prolonged expansion.

Similar to other immortalised erythroid cell lines^19^, SiBBE is a mixed clonal population, revealing a modal chromosome number of 49, XX range 48–50 (50 metaphases). The most common variation were trisomies of chromosome 6, 15 and 19 (34 out of 50), while the rest were other chromosome anomalies including translocations, partial deletions, pseudodicentric and derivative chromosomes (shown in Supplementary Table 1 and Supplementary Fig. 1).

### Further characterisation of SiBBE during differentiation

**Figure 2:**
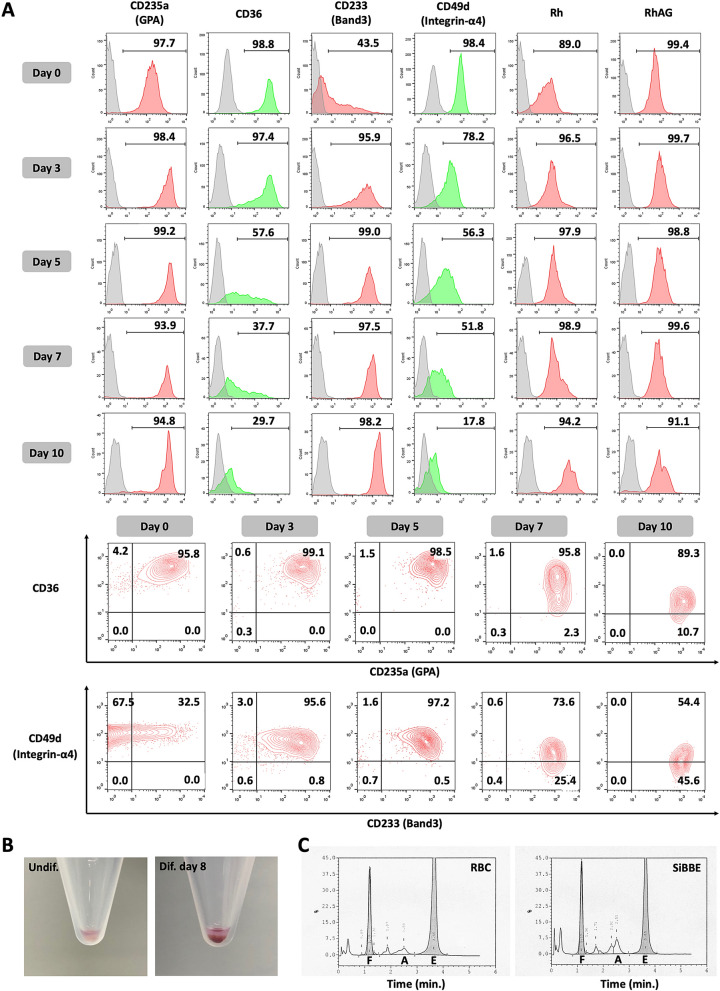
(**A**) Flow cytometric analysis of SiBBE on day 77 in expansion medium showing key erythroid cell marker levels in undifferentiated cells (day 0) and differentiated cells on day 3, 5, 7 and 10 in erythroid differentiation medium. Cells were incubated with antibodies to CD36, glycophorin A, α4 integrin, band3, Rh and RhAG followed by incubation with an IgG1 APC secondary antibody, or with α4 integrin FITC conjugate. The cells were also dual stained by incubation with antibodies to CD36 and GPA or α4 integrin and band 3. (**B**) Pellets of undifferentiated erythroid cells and differentiated erythroid cells on day 8 in culture. (**C**) HPLC traces showing HbF, HbA and HbE levels in the patient RBC and the differentiated cell line (SiBBE).

Flow cytometric analysis was performed with antibodies to key RBC membrane proteins. The undifferentiated cell line (day 77) expressed erythroid markers GPA, CD36, α4-integrin, Rh and RhAG (Fig. 2A, day 0). 41.2 ± 2.4% (n = 3) of cells expressed Band3, which represent the basophilic erythroblast proportion in the population. Following transfer to differentiation media, the level of GPA and Band3 increased whilst the level of CD36 and α4-integrin decreased (Fig. 2A, upper panel) as expected^22^. The level of Rh and RhAG was maintained. To more accurately assess differentiation, the inverse expression of Band 3 vs α4-integrin^19,22^ and GPA vs CD36^19^ was analyzed using dual staining. More than 95% of undifferentiated SiBBE (day 0) expressed GPA^med^CD36^high^ and Band3^low/med^α4-integrin^high^, similar to the profiles of cultured primary pro-/basophilic erythroblasts and the undifferentiated BEL-A line^19,22^. Following transfer to differentiation medium, the expression of GPA and Band3 was inversely related to that of CD36 and α4 integrin, respectively (Fig. 2A, lower panel). At the end of differentiation (day 10), approximately 56.1 ± 9.1% (n = 3) of remaining cells were Band3^high^ α4-integrin^low^, with the remainder having slightly higher levels of α4-integrin, a profile expected for a population of predominately orthochromatic erythroblasts^19,22^, and in line with the morphological analysis (Fig. 1B).

### Globin profile of SiBBE

Before differentiation, the centrifuged SiBBE cells produced pink cell pellets, whereas after differentiation, cells produced red pellets (Fig. 2B), indicating haemoglobin synthesis. Importantly, HPLC analysis showed the differentiated cells expressed a closely similar globin profile to that of the patient RBCs (Fig. 2C). Total Hb level of the patient blood was 5.5 g.dl^−1^ (normal range 12.5–16.8 g.dl^−1^) of which the globin subunit proportions were 24.4% HbF, 5.9% HbA and 69.7% HbE, compared to 29.2% HbF, 9.0% HbA and 61.8% HbE in SiBBE cells. Hence, unlike erythroid cells differentiated from iPSCs of thalassemic patients^12^ the cells maintain expression of adult globin subunits. The line thus presents as an ideal model for evaluating reagents that reactivate foetal globin expression, increasing and normalising total haemaglobin levels whilst in a thalassemic background.

### Increased foetal globin on treatment of SiBBE with hydroxyurea

**Figure 3:**
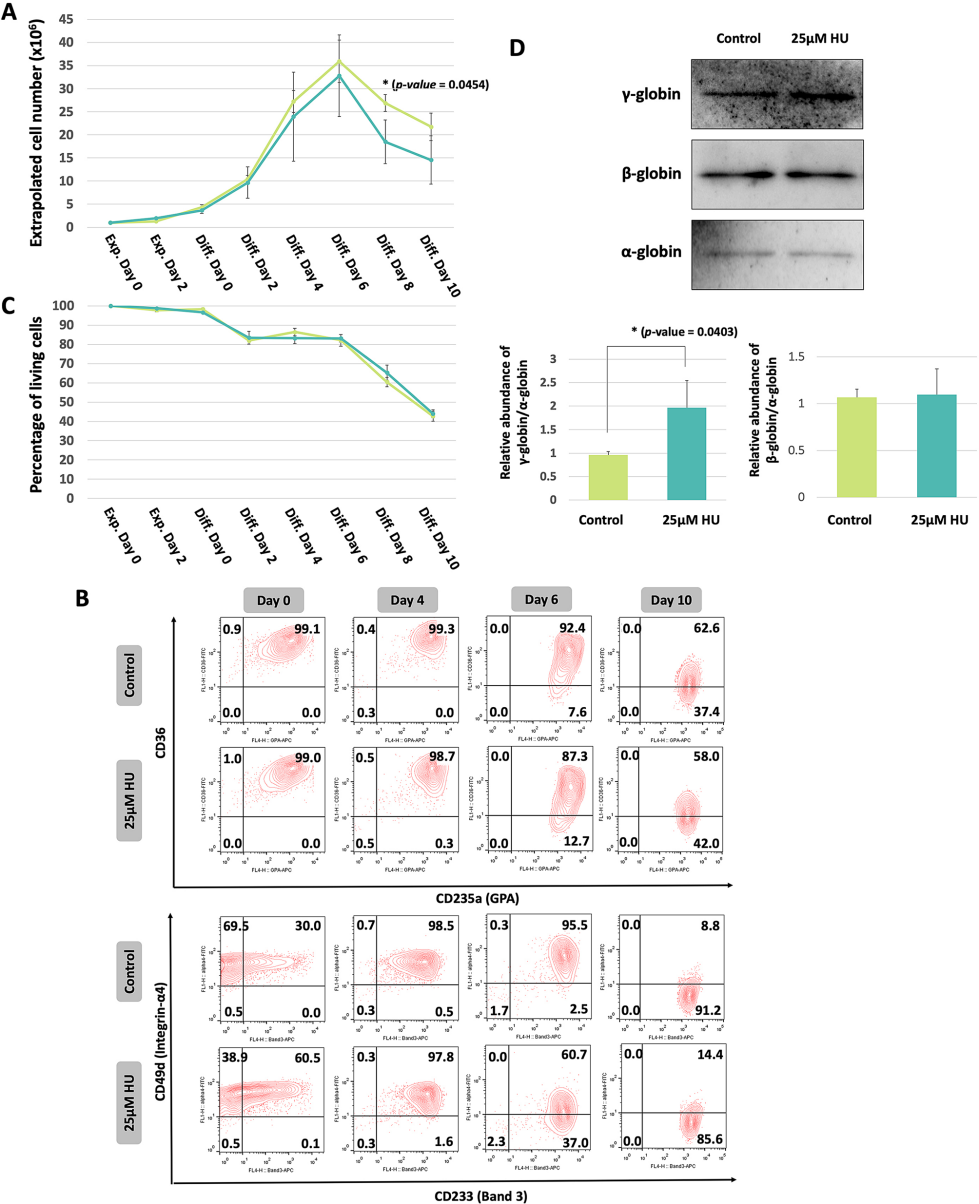
(**A**) Extrapolated cell numbers of HU treated erythroid cells (blue) and control cells (green) (mean ± SD, n = 3). (**B**) Flow cytometric analysis of the cell line showing erythroid differentiation of HU treated erythroid cells and control cells. The cells were dual stained with anti-CD36 or anti-α4 integrin and anti-GPA or anti-band 3. (**C**) Percentage of living cells of HU treated erythroid cells (blue) and control cells (green) determined by Trypan blue assay (mean ± SD, n = 3). (**D**) Gamma and beta globin expression in HU treated erythroid cells (blue) and control cells (green) analysed by Western blot analysis. Alpha globin was used as control. Western blot is representative of 3 repeats. Density of bands on Western blots were quantified using ImageJ (mean ± SD, p < 0.05 by T-test, n = 3). Full-length blots are presented in Supplementary Fig. 4.

To demonstrate the potential of the line as such a screening platform, the cells were treated with hydroxyurea (HU), a drug approved by FDA as a foetal haemoglobin inducer for sickle cell anaemia patients (reviewed by Pule et al.^23^). 25 μM HU were added to the expansion medium for 4 days, and maintained at the same concentration throughout differentiation. There were slightly fewer cells detected in HU treated cultures, but this only reached significance on day 8 of culture (p < 0.05) (Fig. 3A) and may be due to the slightly accelerated differentiation of the HU treated cells; association between reduced proliferation and expedited differentiation of erythroid cells is well documented^24^. Figure 3B shows the inverse expression of CD36 vs GPA and α4-integrin vs Band3, whereby there were more GPA^high^CD36^low^ (12.7% for HU vs 7.6% for control) and Band3^high^ α4-integrin^low^ (37% for HU vs 2.5% for control) cells in the HU treated culture at day 6 than in the untreated control culture. Viability of the cells was unaffected by HU treatment (Fig. 3C). By day 10, HU treated and control cells were at a comparable stage of differentiation and were harvested to determine globin levels by immunoblot. HU treated cells expressed significantly higher γ-globin than controls (Fig. 3D). There was no corresponding decrease in β-globin levels after HU treatment (Fig. 3D), similar to that found in a previous study^19^, likely due to the long half-life and thus persistence of β-globin in cells.

In conclusion, we have generated a model disease erythroid cell line via immortalisation of early erythroid cells differentiated from peripheral blood stem cells of a patient with HbE/β-thalassemia. The cell line recapitulates the globin profile of the patient’s erythroid cells, exhibits a level of ineffective erythropoiesis as found in β-thalassemia^20,21^ and can be induced to increase expression of foetal globin, thus providing a sustainable supply of disease cells for studying pathophysiology of the disease and as a screening platform for novel treatments.

The line has a number of advantages over other models, CD34^+^ cells and iPSCs from β-thalassemia patients, for erythroid culture. Although erythroid cells differentiated in vitro from peripheral blood CD34^+^ cells have a higher expansion rate during differentiation compared to SiBBE using a similar culture system^17^, they undergo a restricted number of cell divisions before terminally differentiating and enucleating, necessitating repeat collection of CD34^+^ cells for repeat cultures. In contrast the unrestricted expansion potential of undifferentiated SiBBE permits a resultant ‘yield’ of differentiated erythroid cells restricted only by volume feasibility and culture cost. This sustainable supply of disease cells also avoids variability introduced due to heterogeneity between patients. The expansion rate of SiBBE during differentiation is in line with that of our immortalised normal erythroid cell lines (BEL-A^19^ and additional lines; paper in preparation). iPSC lines created from β-thalassemia patient cells have the potential to provide a sustainable source of cells, with the reported expansion potential of differentiated erythroid cells from normal iPSC higher than SiBBE but varying widely (33 to 800 fold^25–27^). However, the cells exhibit aberrant or incomplete erythroid differentiation and express predominantly foetal/embryonic rather than adult globin^12,14,15^ making them unsuitable for model lines. Furthermore, iPSC requires complex haematopoietic differentiation systems to obtain haematopoietic stem cells which further limits their practicality; SiBBE already being at an early erythroid cell stage.

## Materials and methods

### Cell isolation and culture

The study was performed in accordance with the Declaration of Helsinki and after approval by the local research ethics committees (The Siriraj Institutional Review Board, SIRB; COA no. SI159/2018). Written informed consent was obtained from a donor who had been diagnosed as HbE/β-thalassemia (HBB: c.9T>C Het, c.79G>A Het (HbE), c.315+16G>C Het, c.316-185C>T Hom; Supplementary Fig. 2). Peripheral blood CD34^+^ cells were isolated from a transfusion dependent HbE/β-thalassemia patient at Siriraj Hospital, Bangkok. Thirty millilitres of blood was diluted with equal volume of Hanks’ Balanced Salt Solution (HBSS) containing 0.6% (v/v) citrate dextrose solution and separated on a Ficoll-Histopaque density gradient at 400*g* for 30 min at 20 °C. The mononuclear cells were harvested and CD34^+^ cells isolated using a MiniMacs direct CD34^+^ progenitor cell isolation kit (Miltenyi Biotec) following the manufacturer’s instructions. The CD34^+^ cells were then immortalised and cultured as described previously^16,17^. Briefly, the isolated cells were maintained in primary medium, which was Basic medium (Iscove’s medium (Biochrom) containing 3% (v/v) human AB serum (Sigma-Aldrich), 2% fetal calf serum (Hyclone, Fisher Scientific), 3 U/ml EPO (Roche), 200 μg/ml transferrin (R&D Systems) and 1 U/ml penicillin/streptomycin (Sigma-Aldrich)) supplemented with 10 ng/ml SCF (R&D Systems) and 1 ng/ml IL-3 (R&D Systems), for 24 h and then transduced with a Tet-inducible HPV-E6/E7 construct. On day 6, the cells were transferred to expansion medium, which was Stemspan SFEM (STEMCELL Technologies) supplemented with 3 U/ml EPO, 10^–6^ M dexamethasone (Sigma-Aldrich), 50 ng/ml SCF and 1 μg/ml doxycycline (Takara Bio), and maintained in this medium thereafter. The cells were counted using trypan blue staining and maintained at density of 2–5 × 10^5^/ml at 37 °C, 5% CO_2_ with total medium changes performed every 2–3 days.

To induce erythroid differentiation, the cultured cells were transferred to primary medium and maintained for 6 days (doxycycline was included from day 0 to day 4). After day 6, the cells were transferred to and maintained in tertiary medium which was Basic medium supplemented with 500 μg/ml transferrin. The cells were counted using trypan blue staining and maintained at 37 °C, 5% CO_2_ with total medium changes performed every other day.

### PCR analysis

400 ng of RNA was reverse transcribed into cDNA using SuperScript III reverse transcriptase (Invitrogen). Primers (Sigma-Aldrich) used were *HPV16 E6* fwd GCGACCCAGAAAGTTACCAC rev GCAACAAGACATACATCGACCGG; *HPV16 E7* fwd GCAACCAGAGACAACTGATCTC rev TGGGGCACACAATTCCTAGTG; *Gamma globin* fwd TGGGTCATTTCACAGAGGAG rev AGACAACCAGGAGCCTTCC.

### Flow cytometry

Aliquots of 2 × 10^5^ cells were washed with 500 μL PBS containing 2 mg.ml^−1^ glucose and 1% BSA (PBS-AG). The cell pellet was resuspended in 50 μL of respective primary antibodies (anti-CD36, anti-α4 integrin FITC conjugates, both from Miltenyi Biotec, anti-CD235a [BRIC256], anti-CD233 [BRIC71], anti-Rh [BRIC69] and anti-RhAG [LA1818] all from IBGRL, Bristol, UK) and incubated for 60 min at 4 °C. The cells were centrifuged at 400*g* and washed once with 500 μL PBS-AG. The cell pellet was resuspended in 50 μL anti-mouse IgG1 APC secondary antibody (Biolegend) and incubated for 30 min at 4 °C, followed by washing as above. For dual staining cells were co-stained with 50 μL of fluorescent-conjugated antibodies and incubated for 30 min at 4 °C, centrifuged at 400*g* and resuspended in 300 μL PBS-AG for analysis on a FACSCalibur (Becton Dickinson).

### SDS-PAGE and Western blot

Proteins were resolved by SDS-PAGE and transferred to PVDF membranes (Millipore) by Western blot. Membranes were blocked with 10% milk powder followed by incubation with primary antibodies (anti-α globin, anti-β globin, anti-γ globin; all Santa Cruz Biotechnology) at 1:2,000 dilution and secondary antibody (rabbit anti-mouse immunoglobulin-HRP; Abcam) at 1:2,000 dilution. Membranes were incubated with Immobilon Crescendo Western HRP substrate (Merck) for 5 min and bands visualised using Image Quant LAS4000 (GE Lifescience).

## Supplementary information

Supplementary file1.

## Data Availability

The data that support the findings of this study and the line is available from the corresponding authors upon reasonable request.

## References

[1] Hirsch RE, Sibmooh N, Fucharoen S, Friedman JM (2017). HbE/beta-thalassemia and oxidative stress: The key to pathophysiological mechanisms and novel therapeutics. Antioxid. Redox Signal..

[2] Cappellini MD, Porter JB, Viprakasit V, Taher AT (2018). A paradigm shift on beta-thalassaemia treatment: How will we manage this old disease with new therapies?. Blood Rev..

[3] de Dreuzy E, Bhukhai K, Leboulch P, Payen E (2016). Current and future alternative therapies for beta-thalassemia major. Biomed. J..

[4] Garner C, Dew TK, Sherwood R, Rees D, Thein SL (2003). Heterocellular hereditary persistence of fetal haemoglobin affects the haematological parameters of beta-thalassaemia trait. Br. J. Haematol..

[5] Skow LC (1983). A mouse model for beta-thalassemia. Cell.

[6] Huo Y, McConnell SC, Ryan TM (2009). Preclinical transfusion-dependent humanized mouse model of beta thalassemia major. Blood.

[7] Doulatov S, Notta F, Laurenti E, Dick JE (2012). Hematopoiesis: A human perspective. Cell Stem Cell.

[8] Ulirsch JC (2014). Altered chromatin occupancy of master regulators underlies evolutionary divergence in the transcriptional landscape of erythroid differentiation. PLoS Genet..

[9] Pishesha N (2014). Transcriptional divergence and conservation of human and mouse erythropoiesis. Proc. Natl. Acad. Sci. U.S.A..

[10] An X (2014). Global transcriptome analyses of human and murine terminal erythroid differentiation. Blood.

[11] Anstee DJ, Gampel A, Toye AM (2012). Ex-vivo generation of human red cells for transfusion. Curr. Opin. Hematol..

[12] Wattanapanitch M (2018). One-step genetic correction of hemoglobin E/beta-thalassemia patient-derived iPSCs by the CRISPR/Cas9 system. Stem Cell Res. Therapy.

[13] Xu P (2015). Both TALENs and CRISPR/Cas9 directly target the HBB IVS2-654 (C > T) mutation in beta-thalassemia-derived iPSCs. Sci. Rep..

[14] Dias J (2011). Generation of red blood cells from human induced pluripotent stem cells. Stem Cells Dev..

[15] Trakarnsanga K (2014). Qualitative and quantitative comparison of the proteome of erythroid cells differentiated from human iPSCs and adult erythroid cells by multiplex TMT labelling and nanoLC-MS/MS. PLoS ONE.

[16] Trakarnsanga K (2017). An immortalized adult human erythroid line facilitates sustainable and scalable generation of functional red cells. Nat. Commun..

[17] Poldee S, Metheetrairut C, Nugoolsuksiri S, Frayne J, Trakarnsanga K (2018). Optimization of an erythroid culture system to reduce the cost of in vitro production of red blood cells. MethodsX.

[18] Kurita R (2013). Establishment of immortalized human erythroid progenitor cell lines able to produce enucleated red blood cells. PLoS ONE.

[19] Daniels DE (2019). Comparing the two leading erythroid lines BEL-A and HUDEP-2. Haematologica.

[20] Mathias LA (2000). Ineffective erythropoiesis in beta-thalassemia major is due to apoptosis at the polychromatophilic normoblast stage. Exp. Hematol..

[21] Arlet JB, Dussiot M, Moura IC, Hermine O, Courtois G (2016). Novel players in beta-thalassemia dyserythropoiesis and new therapeutic strategies. Curr. Opin. Hematol..

[22] Hu J (2013). Isolation and functional characterization of human erythroblasts at distinct stages: Implications for understanding of normal and disordered erythropoiesis in vivo. Blood.

[23] Pule GD, Mowla S, Novitzky N, Wiysonge CS, Wonkam A (2015). A systematic review of known mechanisms of hydroxyurea-induced fetal hemoglobin for treatment of sickle cell disease. Expert Rev. Hematol..

[24] Zermati Y (2000). Transforming growth factor inhibits erythropoiesis by blocking proliferation and accelerating differentiation of erythroid progenitors. Exp. Hematol..

[25] Lapillonne H (2010). Red blood cell generation from human induced pluripotent stem cells: Perspectives for transfusion medicine. Haematologica.

[26] Bernecker C (2019). Enhanced ex vivo generation of erythroid cells from human induced pluripotent stem cells in a simplified cell culture system with low cytokine support. Stem Cells Dev..

[27] Dorn I (2015). Erythroid differentiation of human induced pluripotent stem cells is independent of donor cell type of origin. Haematologica.

