# Supplementary material for: Generation of an immortalised erythroid cell line from haematopoietic stem cells of a haemoglobin E/β-thalassemia patient
Source: Sci Rep. 2020 Oct 8;10:16798. doi: 10.1038/s41598-020-73991-4 (PMC7546635; doi:10.1038/s41598-020-73991-4)
Supplement: Supplementary file 1 — Supplementary file1. [file 41598_2020_73991_MOESM1_ESM.pdf]

GENERATION OF AN IMMORTALISED ERYTHROID CELL LINE FROM HAEMATOPOIETIC STEM CELLS OF A HAEMOGLOBIN E/β-THALASSEMIA PATIENT

Kongtana Trakarnsanga[1,*], Chartsiam Tipgomut[1], Chanatip Metheetrairut[1], Methichit Wattanapanitch[2], Archrob Khuhapinant[3], Saiphon Poldee[1], Ryo Kurita[4], Yukio Nakamura[5], Chatchawan Srisawat[1], Jan Frayne[6,7,*]

[1]Department of Biochemistry, Faculty of Medicine Siriraj Hospital, Mahidol University, Bangkok, Thailand; [2]Siriraj Center for Regenerative Medicine, Research Department, Faculty of Medicine Siriraj Hospital, Mahidol University, Bangkok, Thailand; [3]Division of Hematology, Department of Medicine, Faculty of Medicine Siriraj Hospital, Mahidol University, Bangkok, Thailand; [4]Department of Research and Development, Central Blood Institute, Blood Service Headquarters, Japanese Red Cross Society, Tokyo, Japan; [5]Cell Engineering Division, RIKEN BioResource Research Center, Ibaraki, Japan; [6]School of Biochemistry, University of Bristol, Bristol, UK, [7]NIHR Blood and Transplant Research Unit in Red blood cell products, University of Bristol, Bristol, UK

*Corresponding authors: Kongtana.tra@mahidol.ac.th, Jan.Frayne@bristol.ac.uk

Supplementary Table 1: Detailed karyotypes from 50 metaphases of expanding SiBBE

Karyotypes	Number of metaphases
49,XX,+6,+15,+19	34
48,idem,psu dic(1;20)(q21;q13.1)	1
48,idem,psu dic(1;4)(q21;p16)	1
49,idem,del(11)(p13)	1
49,idem,t(7;13)(q32;p12)	1
48,idem,psu dic(7;19)(q11.2;p13.3)	1
48,idem,psu dic(11;12)(p11.2;p11.2)	1
48,idem,psu dic(4;11)(q23;p13)	1
49,idem,der(6)t(6;?)(p25;?)	1
48,idem,der(18;21)(q10;q10)	1
49,idem,der(13)t(13;?)(q34;?)	1
48,idem,psu dic(2;11)(p13;p11.2)	1
48,idem,psu dic(18;19)(p11.2;p13.1)	1
50,idem,+del(3)(q11.2)	1
49,idem,del(1)(q12),del(18)(p11.2)	1
49,idem,del(9)(p11)	1
48,idem,t(11;19)(q10;q10)	1

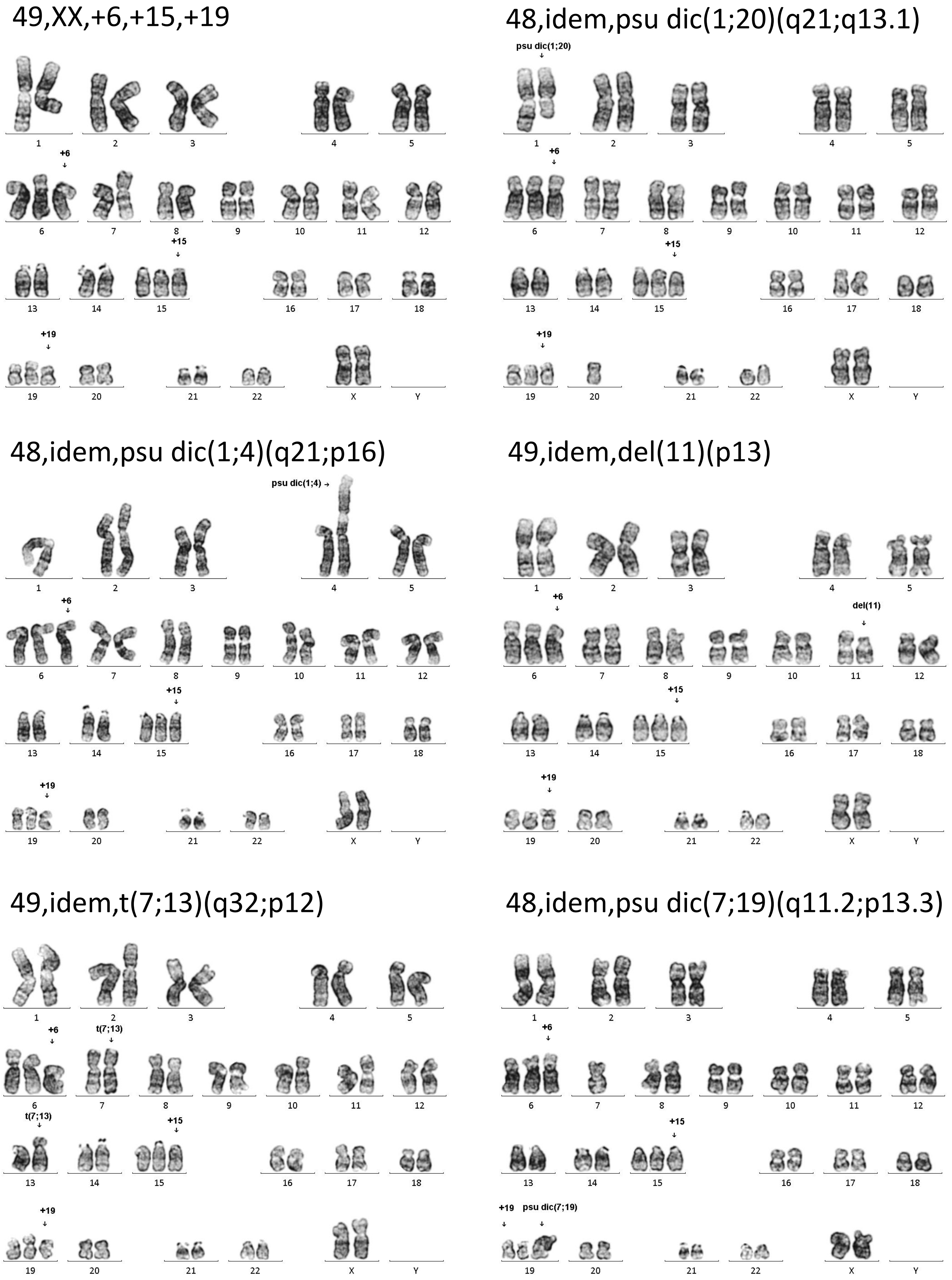

Supplementary Figure 1: G-banded karyotypes of representative metaphases of expanding SiBBE.

48,idem,psu dic(11;12)(p11.2;p11.2)

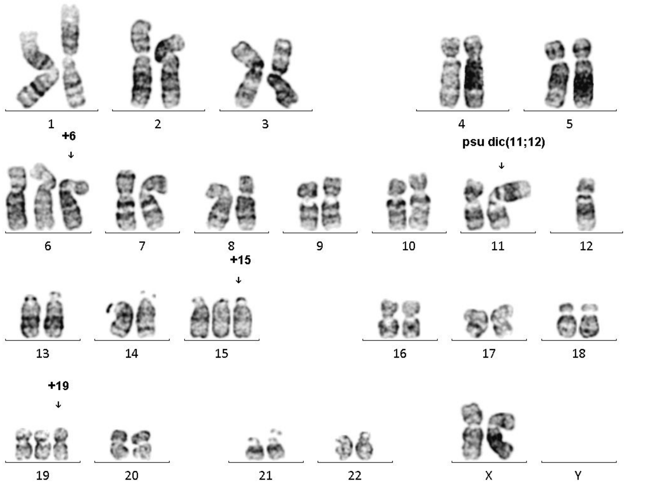

48,idem,psu dic(4;11)(q23;p13)

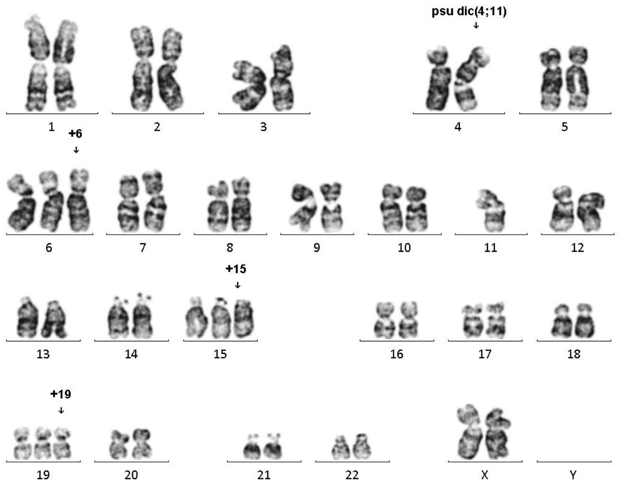

49,idem,der(6)t(6;?)(p25;?)

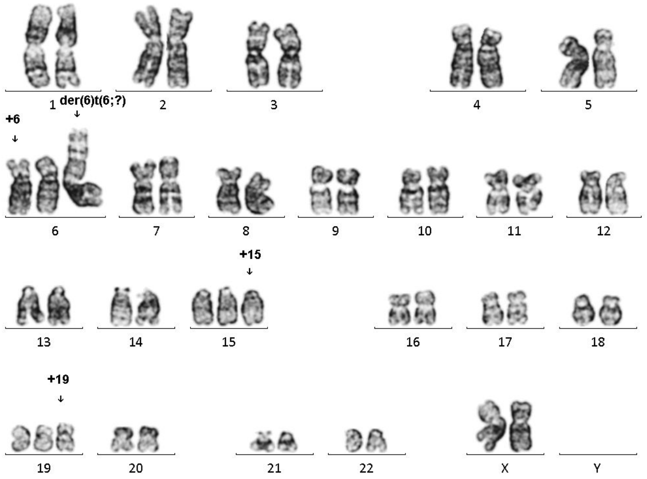

48,idem,der(18;21)(q10;q10)

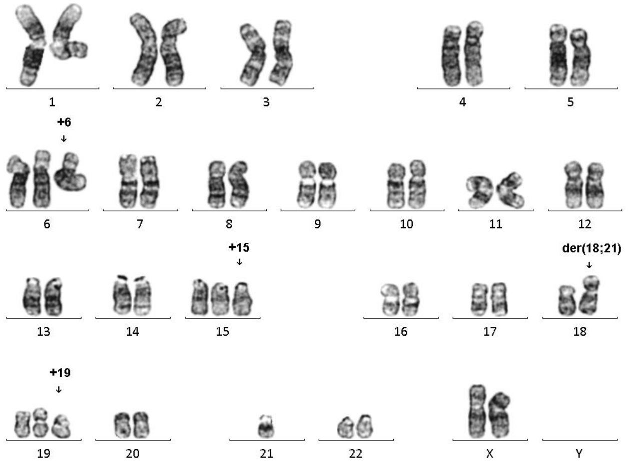

49,idem,der(13)t(13;?)(q34;?)

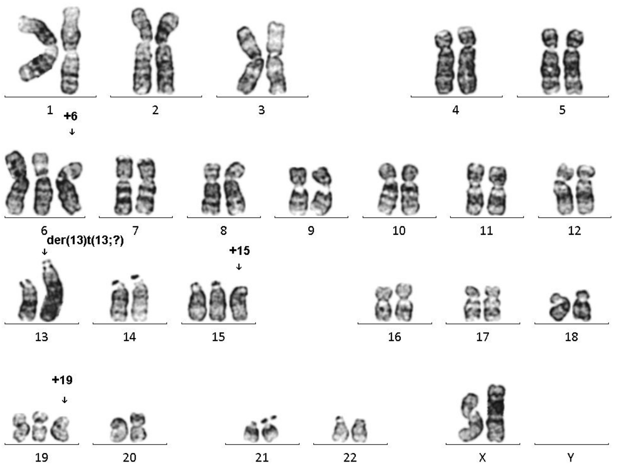

48,idem,psu dic(2;11)(p13;p11.2)

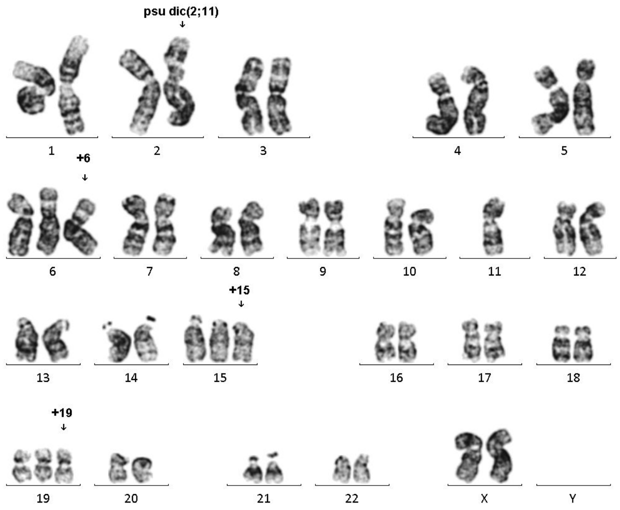

Supplementary Figure 1 (continue)

48,idem,psu dic(18;19)(p11.2;p13.1)

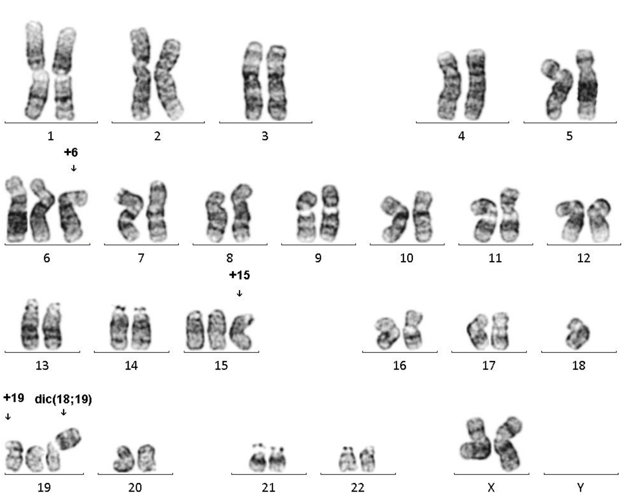

50,idem,+del(3)(q11.2)

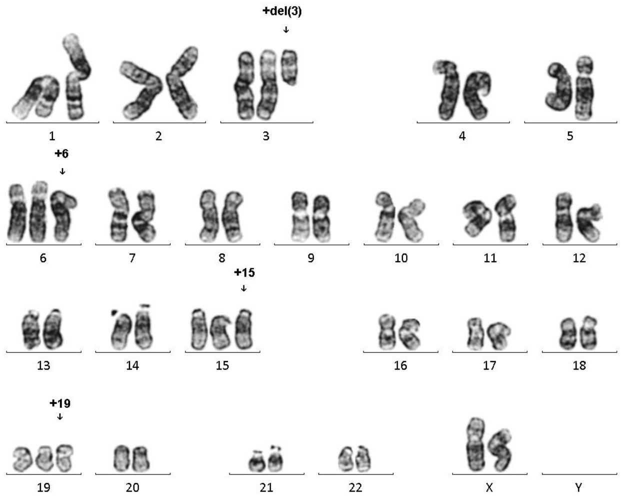

49,idem,del(1)(q12),del(18)(p11.2)

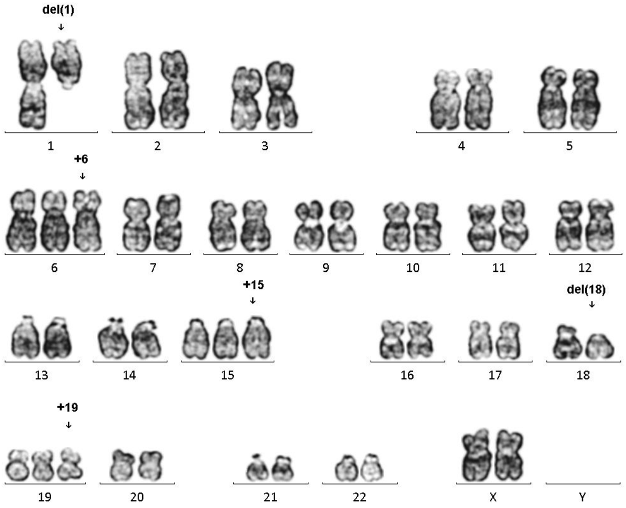

49,idem,del(9)(p11)

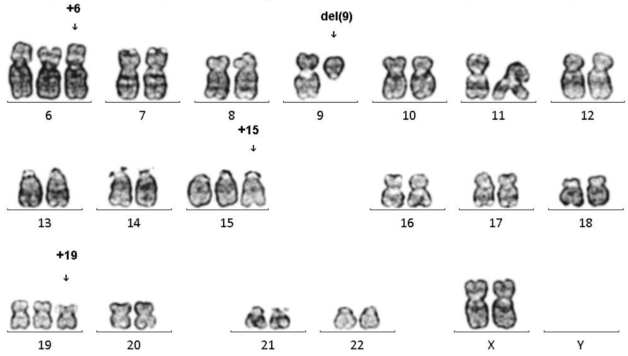

48,idem,t(11;19)(q10;q10)

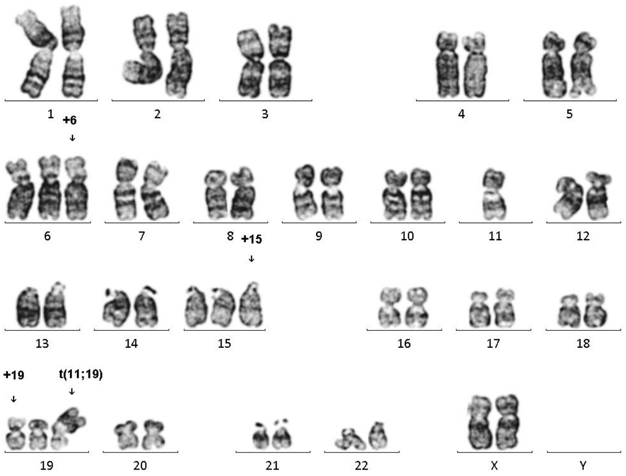

Supplementary Figure 1 (continue)

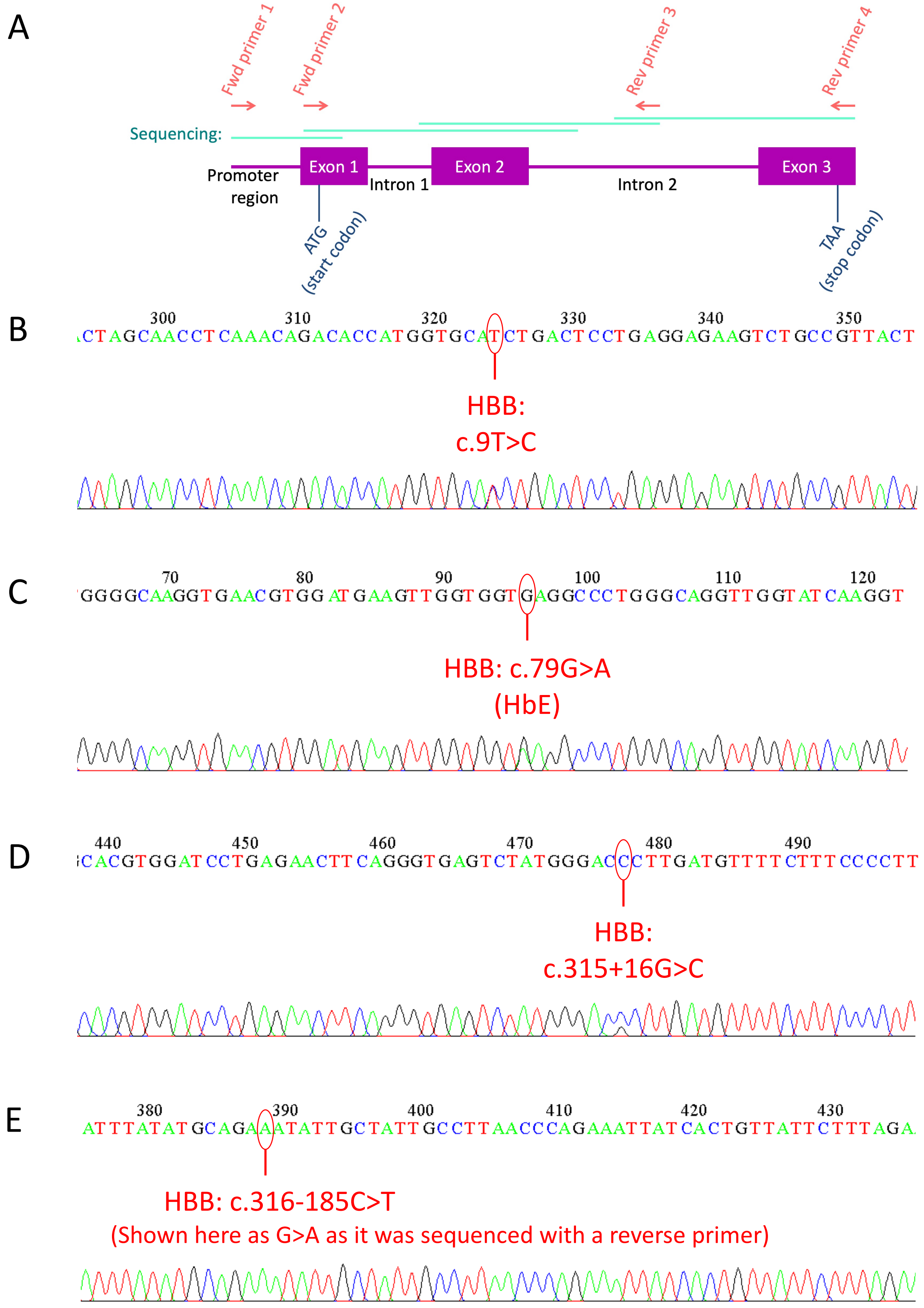

Supplementary Figure 2: Mutations in HBB gene. **A** Beta globin (HBB) gene was amplified from genomic DNA, which was extracted from the patient’s blood, by Phusion High-Fidelity DNA Polymerase (Thermo Scientifics) and sequenced by standard Sanger sequencing. The gene (including exons and introns) was sequenced from 290 nucleotides upstream of the transcriptional start site to 60 nucleotides downstream of the stop codon. **B** heterozygous allele at HBB: c.9T>C in exon 1, **C** heterozygous allele at HBB: c.79G>A in exon 1, creating hemoglobin E (HbE) allele, **D** heterozygous allele at HBB: c.315+16G>C in intron 2, and **E** homozygous mutation HBB: c.316-185C>T in intron 2. Sequencing was performed in the forward direction for **B-D** and in the reverse direction for **E**.

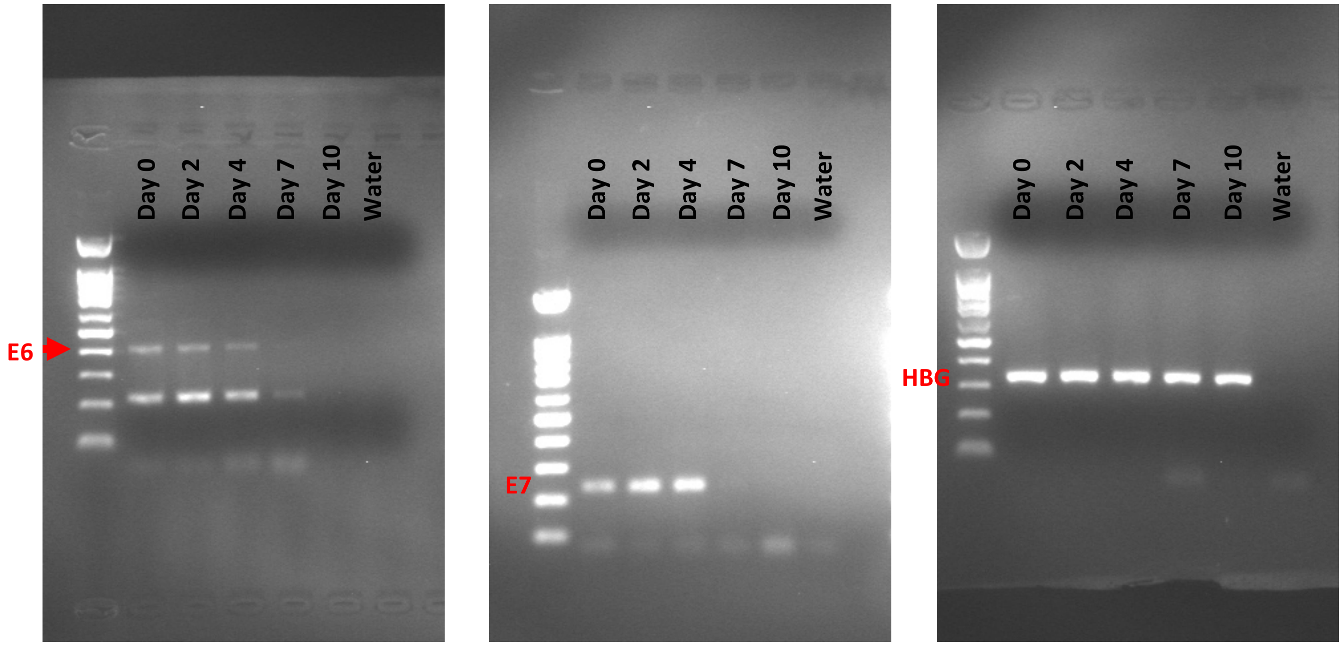

Supplementary Figure 3 Expression of HPV16 E6, E7 and gamma globin (HBG) (control) determined by RT-PCR. Full-length gels of images shown in Figure 1E.

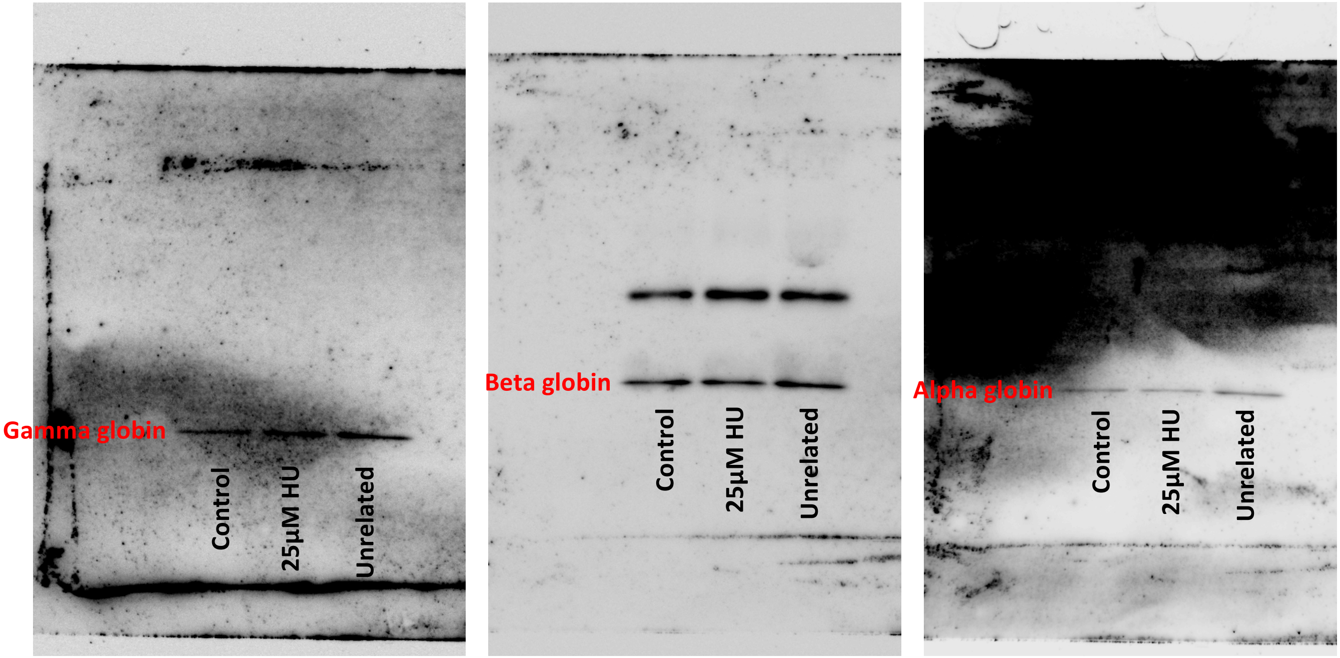

Supplementary Figure 4 Globin isoform expression in HU treated erythroid cells and control cells analysed by Western blot analysis. Full-length blots of images shown in Figure 3D. Unrelated bands represent bands that are not related to this experiment.
